# The Interaction Mechanism Between C14-Polyacetylene Compounds and the Rat TRPA1 Receptor: An In Silico Study

**DOI:** 10.3390/ijms252011290

**Published:** 2024-10-20

**Authors:** Hui Yu, Denghui Gao, Ying Yang, Lu Liu, Xi Zhao, Risong Na

**Affiliations:** 1 College of Science, Beihua University, Jilin 132013, China; huiyu19780413@163.com; 2 National Engineering Laboratory for Druggable Gene and Protein Screening, Northeast Normal University, Changchun 130117, China; gaodh@nenu.edu.cn; 3 College of Plant Protection, Henan Agricultural University, Zhengzhou 450002, China; yangying032@163.com (Y.Y.); nrs@henau.edu.cn (R.N.); 4 Institute of Theoretical Chemistry, College of Chemistry, Jilin University, Changchun 130000, China; l_liu18@mails.jlu.edu.cn

**Keywords:** polyacetylene, TRPA1, cation channel, molecular docking, binding free energy, molecular dynamics simulation

## Abstract

Polyacetylene (PA) compounds, as natural products, exhibit remarkable properties and distinctive chemical activities. Three structurally similar C14-PA compounds—Echinophorin D, Echinophorin B, and Echinophorin A—extracted from plants demonstrate varying biological activities on the Transient Receptor Potential Channel A1 (TRPA1) protein, which belongs to the TRP (Transient Receptor Potential) family. In the current study, we investigated the binding modes of these three PA compounds with TRPA1 using molecular dynamics (MD), molecular docking, binding free energy calculations, and quantum mechanics/molecular mechanics (QM/MM) methods. Initially, a putative binding site (site-II) in TRPA1 was identified for these compounds; Echinophorin B was found to stabilize the upward A-loop of TRPA1, which is critical for its activation. Furthermore, the binding affinity calculations of PA compounds through molecular fragment decomposition indicate that the arrangement of two triple bonds and one double bond in C14-PA compounds is vital for regulating TRPA1 bioactivity. Additionally, the lipophilic and electronic properties of the three molecules were analyzed in relation to binding affinity, establishing a correlation between TRPA1 activity and these molecular properties.

## 1. Introduction

**Figure 1:**
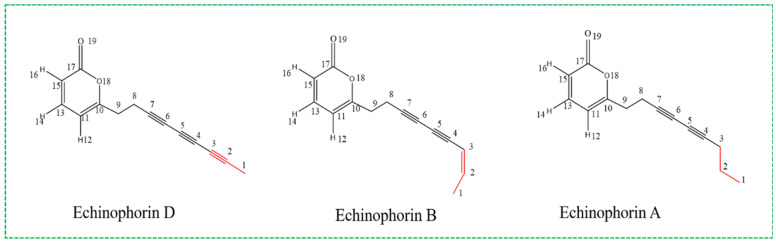
Chemical structure diagram of C14-PA compounds.

Polyacetylenes (PAs) and their derivatives are a class of chemical substances and secondary metabolites commonly found in Asteraceae and Umbelliferae plants [1]. These compounds typically contain at least two conjugated carbon–carbon triple bonds and exhibit a wide range of biological activities. The most prevalent PAs are C17 derivatives, such as cicutoxin, which is found in water hemlock (Cicuta virosa) and binds to the GABA receptor (γ-aminobutyric acid receptor), thereby blocking GABAergic responses [2,3]. Additionally, falcarinol and falcarindiol demonstrate anti-cancer and anti-inflammatory properties at non-toxic concentrations for humans [4]. In 2018, Giuseppina Chanese et al. extracted three types of C14-polyacetylenes from the Echinophora platyloba plant, designated Echinophorin D (3), Echinophorin B (4), and Echinophorin A (5). A structural diagram is presented in Figure 1. The authors evaluated the biomedical significance of these three PA compounds on rat TRP channels (TRPA1, TRPV1, TRPV2, TRPV3, TRPV4, and TRPM8) using experimental methods. The results indicated that the C14-PA compounds effectively regulated TRPA1, while their overall activity on other TRP channels was negligible [5]. Through a fluorimetric test, they observed that rat TRPA1-HEK293 cells exhibited an increase in intracellular [Ca^2+^]i upon the application of compounds **3**–**5**. The results are summarized in Table S1. TRPA1 is associated with a range of pathological conditions, including pain, skin diseases, pulmonary diseases, urogenital system disorders, and gastrointestinal diseases. It is a pivotal player in neuropathic and inflammatory pain and is extensively investigated as a target for the development of novel analgesic and anti-inflammatory agents [6]. Furthermore, TRPA1 may modulate specific neural circuits, be linked to comorbid depression, and impact synaptic plasticity. The regulation of TRPA1 by PA compounds represents a potential mechanism to explore for analgesic, anti-inflammatory, and antidepressant effects.

**Figure 2:**
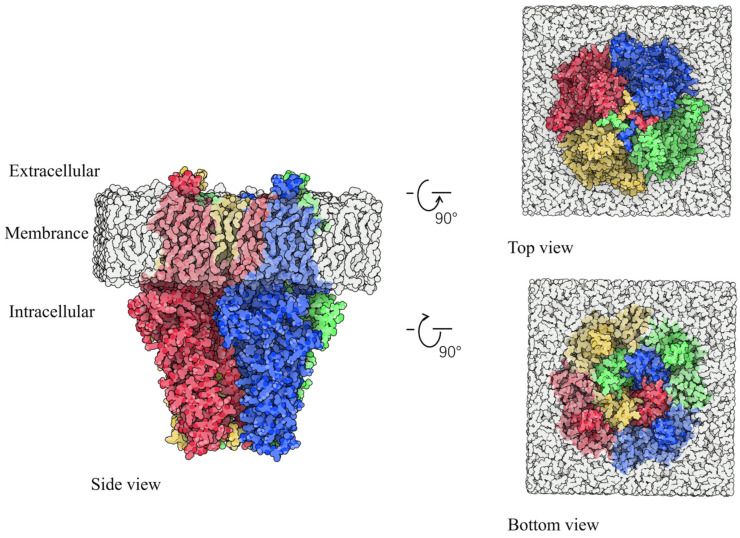
The 3D conformation of TRPA1. Blue, green, red, and yellow are the A, B, C, and D chains of TRPA1. White represents the phospholipid layer.

The TRP channel superfamily represents a complex and ancient system, widely distributed across various organs and tissues in the human body, playing a crucial role in regulating cellular functions. David Julius elucidated the mechanisms of pain through his studies of natural plants, such as peppers and mint, leading to the identification of TRPV1, TRPM8, and TRPA1 [7]. To date, TRP channels have been identified in over 30 mammalian species [8]. Based on genetic homology, these channels are generally classified into six subfamilies: TRPA (ankyrin), TRPV (vanilloid), TRPC (canonical), TRPM (melastatin), TRPP (polycystin), and TRPML (mucolipin) [9]. The TRP channel protein forms a tetramer composed of four subunits, with each subfamily exhibiting multiple subtypes due to variations in these subunits. TRP receptors of different subtypes are expressed in distinct organs and tissues, fulfilling various physiological roles; for example, the TRPV family includes TRPV1-V6 and M8 [10], while the TRPC family encompasses C1-C7 [11]. TRPA1, a member of the TRPA family, is a potent non-selective TRP cationic T-type channel permeable to sodium, potassium, and calcium ions. In 2003, the expression of TRPA1 in dorsal root ganglion and trigeminal ganglion neurons was observed in mice [12]. Human TRPA1 consists of 1119 amino acids, whereas the rat variant contains 1125 amino acids. Figure S1 illustrates the amino acid sequences of human TRPA1 (residues 447–1078) and rat TRPA1 (residues 447–1081). The sequence similarity between rat and human TRPA1 is substantial (percent similarity: 78.27), indicating that studies on rat TRPA1 can enhance our understanding of human TRPA1. Figure 2 presents the three-dimensional configuration of rat TRPA1.

Studies have demonstrated that TRPA1 functions as a cold sensory channel, exhibiting heightened sensitivity to both endogenous ligands and exogenous chemical stimuli [7,13,14]. Upon activation, TRPA1 facilitates an increase in extracellular Ca^2+^ influx, subsequently regulating a range of physiological responses [12]. In recent years, significant attention has been directed toward the structural characterization of TRPA1, as well as the binding patterns of its agonists and antagonists. TRPA1 agonists are classified into covalent [7] and non-covalent [15] categories based on their mechanisms of action. Covalent agonists include electrophilic compounds such as allyl isothiocyanate (AITC), acrolein, and 4-hydroxynonenal, which covalently modify specific cysteine residues within the intracellular N-terminal region of TRPA1. In 2020, Yang Suo et al. reported on the cryogenic electron microscopy structure of a TRPA1 ion channel complexed with the irreversible covalent agonist JT010 and the reversible covalent agonist benzyl isothiocyanate (BITC) [16]. Additionally, Jianhua Zhao et al. reported on the structure of TRPA1 in complex with the irreversible electrophilic agonist iodoacetamide (IA) and the small-molecule antagonist A-967079 [17]. Non-covalent agonists encompass compounds such as carvacrol, menthol, clotrimazole, propofol, and piperidine carboxamides [18]. In 2021, Chang Liu proposed a binding mechanism between the non-covalent agonist GNE551 and TRPA1 [19]. These studies provide valuable insights into the binding sites of other compounds. Recently, considerable attention has been devoted to the physiological processes of functional TRP channels, which have also been explored for drug discovery aimed at treating neuropathic chronic pain. Compared to known regulatory small molecules, the natural extraction and widespread distribution of PA compounds present unique advantages. Moreover, the regulation of TRP channels by PA natural products marks a novel contribution to the field. Therefore, investigating the interaction between C14-PA compounds and TRPA1 holds significant importance. Previous studies on PA compounds have predominantly concentrated on their separation, extraction, and the observation of certain biological activities; however, there is a notable scarcity of theoretical investigations into their mechanisms of action. Currently, the three-dimensional conformation of TRPA1 is well defined, facilitating molecular simulation analyses of PA compounds interacting with TRPA1. In this study, three PA compounds extracted by Giuseppina Chanese are selected for a comprehensive computational investigation that includes molecular docking, molecular dynamics simulation, linear interaction energy (LIE) analysis, interaction analysis, and molecular property assessment. Our objective is to elucidate the binding behavior of C14-PA compounds with the TRPA1 channel protein, with the aim of providing a theoretical foundation for the screening and discovery of novel polyacetylene scaffold compounds that target the TRPA1 channel.

## 2. Results

### 2.1. Identification of Binding Sites

**Figure 3:**
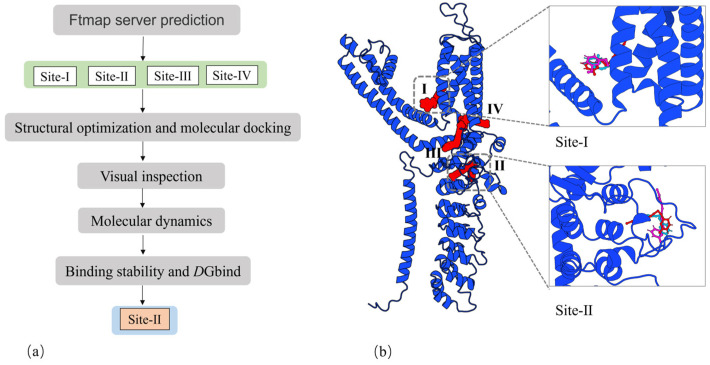
(**a**) A flow chart of the process for determining the potential binding site. (**b**) Possible binding sites of TRPA1.

**Figure 4:**
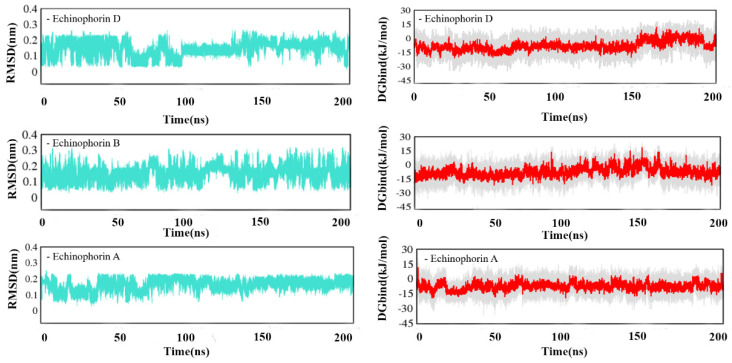
The RMSD and *D*Gbind of Echinophorin D, Echinophorin B, Echinophorin A, and TRPA1 at the binding site changes with time at site-Ⅱ.

A suitable 3D conformation of rat TRPA1 was constructed using the SWISS-Model server. The Ftmap server predicted the potential binding sites for the three PA compounds. Among these predictions, one site was situated in the membrane (site-I, Figure 3), while the others were located at the lower boundary of the membrane. Chang Liu et al. have suggested that site-I is the binding site for non-covalent agonist GNE551 in TRPA1. Site-Ⅱ (Figure 4) was identified in the A-loop flexible region, and the covalent sites beneath this region have been the subject of extensive research. The A-loop region is situated between β1.2 and H5 (Figure S1, Residue ID: 660–690). Site-Ⅳ is occupied by calcium ions in the natural biological environment. We conducted molecular docking and molecular dynamics simulations for each site. Our analysis of the structural trajectories revealed that the three PA compounds could stably reside at site-Ⅰ and site-Ⅱ. However, at site-Ⅲ and site-Ⅳ, the ligands tended to dissociate from the predicted binding cavities. Consequently, we focused our attention on site-Ⅰ and site-Ⅱ, as detailed in reference [20].

The green curves in Figure S2 and Figure 4 represent the root mean square deviation (RMSD) for the three PA compounds at site-Ⅰ and site-Ⅱ. As can be seen from the RMSD diagrams, small molecules exhibit less fluctuation at the binding sites. Next, the relative binding free energy (*D*Gbind) was calculated for the three PA compounds bound to site-Ⅰ and site-Ⅱ. Figure S2 and Figure 4 illustrate the relationship between the relative binding free energy and the simulation time at sites Ⅰ and Ⅱ (γ = 0). In Figure S2, although the fluctuation in small molecules at site-Ⅰ is small, the *D*Gbind values represented by the red curve are all above 0 KJ mol^−1^, indicating weak binding affinity. Conversely, *D*Gbind values at site-Ⅱ suggest strong binding affinity.

To further validate that site-II is the binding site, a relatively stable dynamic trajectory was selected for the calculation of Δ*G*_bind_, and the results are presented in Table 1. In this calculation, U^vdw^ represents the average vdW energy, and U^ele^ represents the average electrostatic energy. Δ*G*_bind_ was calculated using the LIE method, as per Equation (1), with γ/Δ*G*_bind_ representing the percentage of the γ term associated with hydrophobic properties in the calculated binding free energy. The Δ*G*_bind_ values for Echinophorin D, Echinophorin B, and Echinophorin A are −24.506, −24.612, and −24.566 KJ/mol, respectively. These values are consistent with the experimental results (ΔG^EXP^, Table 1) [5]. Consequently, we believe that site-II is a reliable binding site. Additionally, site-II is also identified as the binding site for AITC, JT010, BITC, and IA to TRPA1 receptors. The difference lies in the fact that AITC, JT010, BITC, and IA are bound covalently.

### 2.2. Interaction Analysis

**Figure 5:**
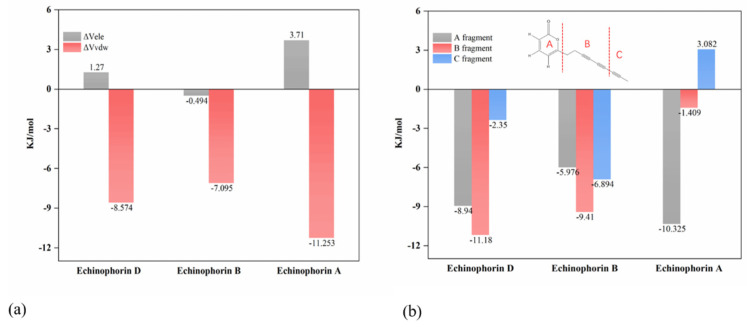
(**a**) At site-Ⅱ, Coulomb and van der Waals interactions between three PA compounds with TRPA1. (**b**) Binding energy of small molecular fragments with proteins.

**Figure 6:**
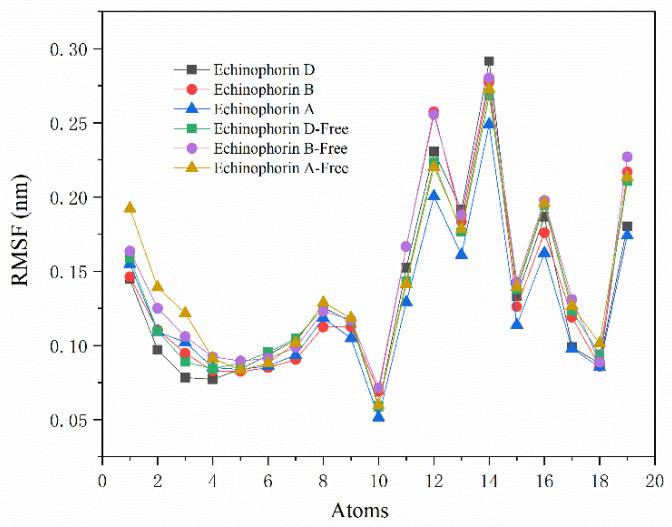
An RMSF diagram of the three PA compounds. Echinophorin D represents the RMSF of the ligand in the bound state. Echinophorin D-Free represents the RMSF of the ligand in the free state.

Figure 5a illustrates the values of ΔV_ele_ and ΔV_vdw_, indicating that ΔV_vdw_ contributes more significantly to the binding energy. Specifically, ΔV_vdw_ plays a dominant role in the binding process. Interestingly, Echinophorin B exhibits weak electrostatic interactions with the surrounding residues, whereas Echinophorin A and Echinophorin D demonstrate repulsive electrostatic interactions that adversely affect the binding energy. To further investigate the influence of structural changes in small molecules on binding affinity, the molecule was divided into three fragments, as shown in Figure 5b. The binding affinities of these fragments with TRPA1 were calculated individually. Fragment A is α-pyranone, fragment B contains two carbon–carbon triple bonds, and fragment C comprises a different moiety. In Echinophorin B, fragments A, B, and C exhibit strong interactions with TRPA1. In Echinophorin D, the interactions between fragments A and B with TRPA1 are enhanced compared to those in Echinophorin B; however, the interaction of fragment C is significantly weakened. Interestingly, in the compound Echinophorin A, fragment A maintains a strong interaction with TRPA1, while the interaction of fragment B is significantly weakened, and fragment C does not facilitate binding. Notably, Echinophorin A has one more rotatable bond than Echinophorin B and Echinophorin D. This substantial difference in fragment C may be attributed to the greater conformational flexibility of single bonds compared to double and triple bonds. To investigate this further, we conducted root mean square fluctuation (RMSF) analysis. Figure 6 illustrates the conformational flexibility of the three PA compounds at the binding site. The RMSF in the free state is significantly higher than that in the bound state. Additionally, it is evident that the RMSF of Echinophorin A at the end of the straight chain (atoms 1–3) is markedly larger than that of Echinophorin B and Echinophorin D.

**Figure 7:**
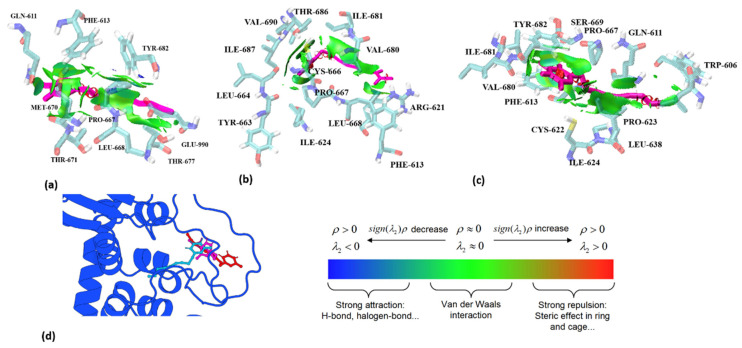
The analysis of weak interactions between three PA compounds and TRPA1 complexes at site-II is presented. The PA molecules are depicted as purple sticks, while the surrounding residues are represented as blue sticks. Specifically, (**a**) illustrates Echinophorin D, (**b**) depicts Echinophorin B, and (**c**) shows Echinophorin A. In panels (**a**–**c**), blue surfaces denote hydrogen bonding, green surfaces indicate van der Waals interactions, and red surfaces represent repulsion. Panel (**d**) illustrates the binding conformations of the small molecules, where the red stick represents Echinophorin D, the pink stick represents Echinophorin B, and the sky-blue stick represents Echinophorin A.

Further observations of the binding conformations of the three PA compounds reveal interesting insights. Figure 7d presents a conformational snapshot of the three small molecules within the simulated trajectory of the active site. The conformational differences at the α-pyrone terminal are minimal. Notably, the molecules with triple and double bonds at the end of the branched chain align in the same direction, while the ligand molecule with a single bond faces the opposite direction. Combining fragment decomposition with RMSF analysis, we propose that the difference in the binding conformation of Echinophorin A arises from the ease of conformational changes associated with the carbon–carbon single bond. This conformational flip occurs when the docking position does not satisfy the principle of geometric complementarity. In summary, three key conclusions can be drawn: (1) In the molecular dynamics simulations involving the three ligands and proteins, the interaction residues and binding positions of the α-pyranone terminal are similar. This suggests that the α-pyranone segment plays a significant role in the binding of TRPA1. (2) The carbon–carbon double bond at the end of the straight chain exhibits a stronger binding effect than the carbon–carbon triple bond. (3) The terminal carbon–carbon single bonds in straight chains can easily lead to drastic changes in binding conformation.

**Figure 8:**
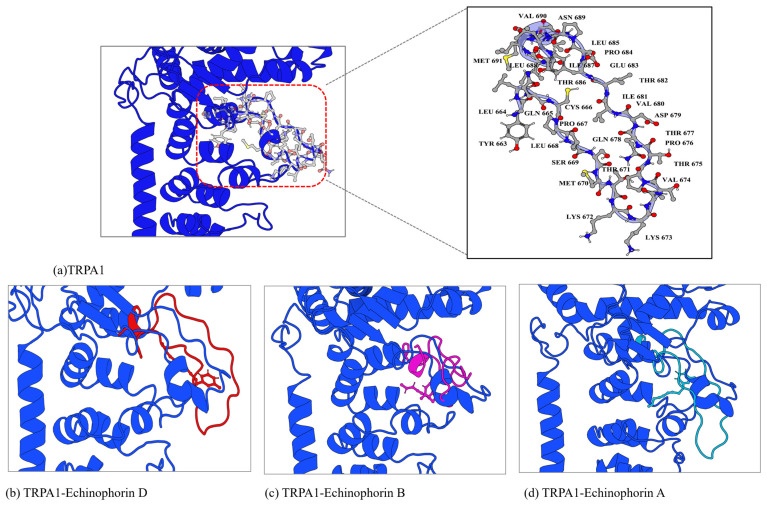
The conformational changes in binding site-II following molecular dynamics simulations are illustrated here. The blue cartoon represents the conformation of the A-chain of TRPA1 (PDB code: 6V9W) in its unbound state. The ball-and-stick model depicts the residues in the A-loop region. The red ligand, Echinophorin D, is shown alongside a corresponding red loop, illustrating how bound Echinophorin D influences the A-loop conformation during the simulation. Similarly, the pink representation corresponds to Echinophorin B, with its associated pink loop demonstrating the impact of bound Echinophorin B on the A-loop conformation. Lastly, the green representation indicates Echinophorin A, accompanied by a green loop that highlights how bound Echinophorin A affects the A-loop conformation throughout the simulation.

The analysis of weak interactions, including hydrogen bonds and van der Waals (vdW) forces, enhances our understanding of ligand–receptor interactions. The dynamic analysis results of these interactions were visualized using aRDG. The calculated weak interactions between the three PA compounds and TRPA1 are presented in Figure 7. The predominant surface color of the three PA compounds and TRPA1 is green, further indicating that van der Waals interactions play a significant role in binding. The differences in the green areas of (a), (b), and (c) reflect variations in their binding affinities. In panel (a), Echinophorin D interacts with the surrounding residues GLN-611, PHE-613, TYR-682, GLU-990, THR-677, LEU-668, PRO-667, MET-670, and THR-671. The residue numbers are based on homologous modeling programs, as detailed in Supplementary Figure S1. In panel (b), Echinophorin B interacts with the surrounding residues TYR-663, LEU-664, ILE-687, VAL-690, THR-686, ILE-681, VAL-680, ARG-621, PHE-613, ILE-624, LEU-668, PRO-667, and CYS-666. Notably, the three PA compounds primarily interact with the same residues in the binding site, as summarized in Table 2. The branches of the three PA compounds exhibit strong van der Waals interactions with the nonpolar residues ILE-681 and VAL-680. In contrast, the branched ends of the compounds demonstrate weaker van der Waals interactions with PHE-613, ARG-621, and LEU-668. The amino acids surrounding α-pyrone all exhibit some degree of van der Waals interactions, with the weak cyan color indicating insufficient hydrogen bonding capability. Additionally, it is noteworthy that only compound B was able to stabilize the upward conformation of the A-loop in the simulation results, as shown in Figure 8. Previous studies have reported that the dynamic activation loop (A-loop) adopts a downward conformation in the agonist-free channel, partially occluding a reactive pocket containing C621 (C622). When the covalent agonist binds to C621, the A-loop transitions to an upward conformation, facilitating the entry of C665 (C666) into the reaction pocket [17]. Additionally, we discovered that compound B interacts with both C622 and C666 at the binding site. This observation suggests that the stabilization of the upward deflection of the A-loop is not exclusive to covalent agonists.

In panel (c), Echinophorin A interacts with the surrounding residues ILE-624, CYS-622, PHE-613, VAL-680, ILE-681, TYR-682, SER-669, PRO-667, GLN-611, TRP-606, PRO-623, and LEU-638. Additionally, α-pyrone exhibits strong van der Waals interactions with the residues PHE-613, VAL-680, ILE-681, TYR-682, SER-669, and PRO-667. Furthermore, the branches of the three PA compounds interact with the residues GLN-611 and PRO-623, with minimal interaction observed at the branched ends. Through interaction analysis, we determined that the binding of the three polyacetylene molecules at site-II primarily relies on van der Waals interactions. An examination of the surrounding residues revealed that most are nonpolar, with very few charged amino acids present. The abundance of nonpolar residues suggests the formation of a hydrophobic pocket. The nonpolar regions of the small molecules are stably bound to the nonpolar surfaces of the protein due to the phenomenon of desolvation within the pocket. Additionally, we observed that there are minimal hydrogen bonds between the three PA compounds and the surrounding residues. The interaction analysis indicates that the attraction of the three polyacetylene (PA) compounds to the surrounding nonpolar residues primarily occurs through van der Waals dispersion interactions, while electrostatic interactions are minimal. This finding aligns with the energy decomposition results calculated using the Linear Interaction Energy (LIE) method, as illustrated in Figure 5a.

### 2.3. Molecular Properties

**Figure 9:**
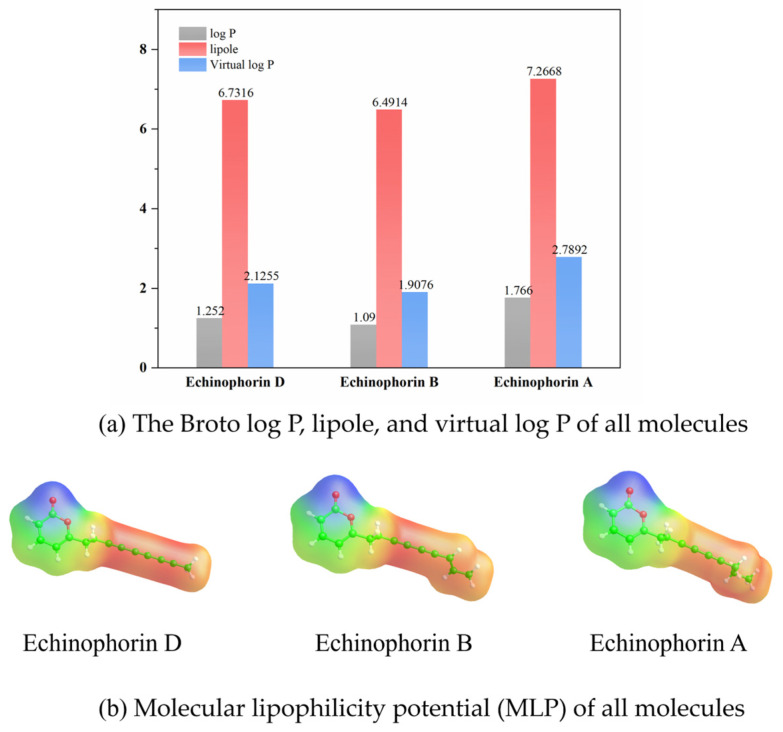
Calculation results of lipid properties of Echinophorin D, Echinophorin B, and Echinophorin A molecules.

The differences in binding affinity and binding modes among the three polyacetylene (PA) compounds with similar structures have piqued our interest in their structure–property relationships. For drug molecules, key properties include electron distribution and lipophilicity, both of which are closely related to ligand–protein binding affinity. To assess the hydrophobic properties of the three PA compounds, we obtained the Broto log P, virtual log P, lipole, and molecular lipophilic potential (MLP) using VegaZZ software. These properties were calculated based on stable conformations, as illustrated in Figure 9. The red regions indicate areas of higher lipophilicity, while the blue regions signify lower lipophilicity in the molecular lipophilic potential (MLP). An analysis of the MLP reveals that the three polyacetylene (PA) compounds exhibit strong hydrophobicity, which is a primary factor contributing to their stable binding within hydrophobic pockets. The values of log P, lipole, and virtual log P clearly demonstrate that Echinophorin A has the highest lipophilicity compared to the other two compounds. This increase may be attributed to the carbon–carbon bond modification at the end of the branched chain, which enhances the lipophilicity of the Echinophorin A molecule. In comparison to the binding conformations of the three PA compounds, the pronounced hydrophobicity of Echinophorin A appears to be a significant contributing factor.

**Figure 10:**
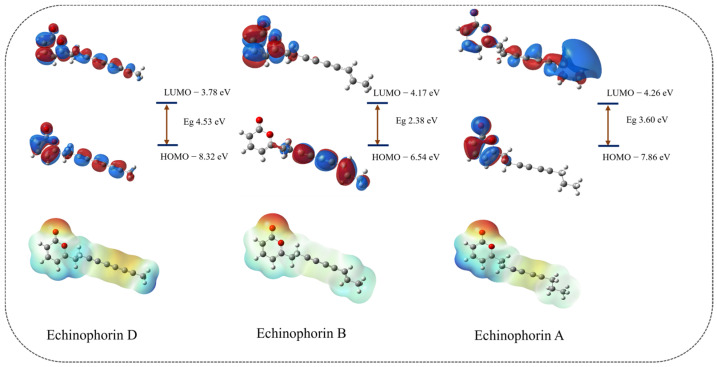
The HOMO, LUMO, and EPS in the background charge of the protein.

For electronic properties, we consider the cases with and without background charges separately. The orbital and electron distributions of the three polyacetylene (PA) compounds are influenced by the potential charge fields (PCFs) provided by the surrounding residues. Notably, the significant difference in the orbital distribution of Echinophorin A compared to Echinophorin D and Echinophorin B may be attributed to variations in the binding environment. The combination of Echinophorin D and Echinophorin B, which share similar environments, warrants further attention. The electrostatic potential surfaces (EPSs) of the three PA compounds are illustrated in Figure S3 and Figure 10. The electrostatic potential value of the red region is negative, indicating that this area is more likely to donate electrons and is thus more nucleophilic than other regions. Conversely, the blue area exhibits a positive electrostatic potential, suggesting that it is more prone to accept electrons and is therefore more electrophilic than other areas. While the electrostatic potential surfaces (EPSs) indicate that the small molecule is electrophilic, the interaction analysis reveals that the electrostatic interactions at the binding site are very weak. Based on the amino acids surrounding the site, we hypothesize that the weak Coulomb effect is attributed to the non-electrostatic environment created by the surrounding uncharged amino acids. The electrostatic potential (ESP) diagram reveals that the electrostatic potential of the three polyacetylene (PA) compounds underwent changes. These changes in electrostatic potential surfaces (EPSs) are primarily due to the presence of polar amino acids near the ligand. The influence of background charges on the three PA compounds helps to elucidate the differences in their electrostatic interactions.

Simultaneously, we calculated the εHOMO (eV) and εLUMO (eV), with the results presented in Table 3. Additionally, we computed the vertical ionization potential (VIP) and vertical electron affinity (VEA). VIP represents the energy required to ionize the first valence electron from a small molecule, while VEA denotes the energy required to stably bind additional electrons. The frontier orbital energies, εHOMO and εLUMO, are important parameters of molecular electronic structure. A more negative εHOMO value indicates a weaker ability to donate electrons [21], whereas a more negative εLUMO value suggests a greater likelihood of accepting electrons. Under the influence of background charges, the orbital distribution exhibits significant changes, as illustrated in Figure 10. The HOMO electron cloud of Echinophorin D is noticeably deformed, likely due to the significant influence of the potential charge fields (PCFs) on the delocalized π bonds of the carbon–carbon triple bond. Echinophorin B exhibits smaller vertical electron affinity (VEA) and vertical ionization potential (VIP) values compared to Echinophorin D. Our analysis of molecular properties indicates that the primary difference between Echinophorin D and Echinophorin B lies in their electronic properties, which result from alterations in the carbon–carbon bonds.

**Figure 11:**
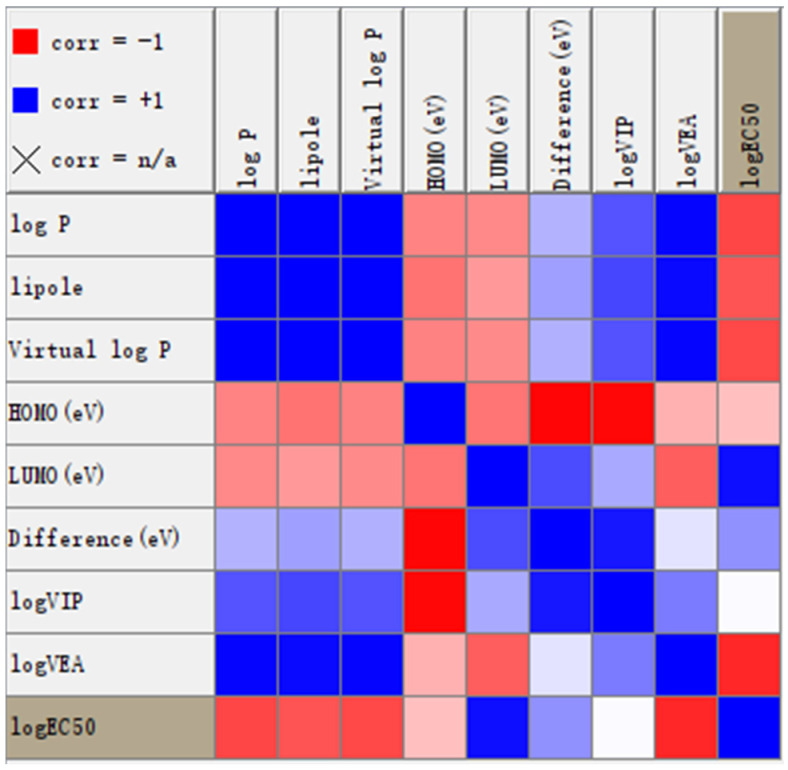
Correlation matrix diagram between Ec50 and molecular properties. The deeper the red color, the stronger the negative correlation; the deeper the blue color, the stronger the positive correlation.

The KNIME nodes for linear correlation and Weka (Linear Regression 3.7) were utilized to examine the linear relationships between biological activity and various properties. The correlation matrix is presented in Figure 11. LogEC50 is positively correlated with εLUMO and negatively correlated with log P and vertical electron affinity (VEA). The linear regression model is expressed as logEC50 = (−0.0321) × logP + (0.259) × LUMOeV + (−0.2866) × logVEA + 3.0105.

## 3. Discussion

In this paper, we found that C14-PA compounds predominantly bind to site-II of rat TRPA1 via van der Waals interactions. At this site, the small molecule exhibits a relatively stable root mean square deviation (RMSD) and a low root mean square fluctuation (RMSF), indicating minimal conformational fluctuations. The presence of two triple bonds and one double bond stabilizes the upward conformation of the A-loop. In C14-PA compounds, changes in activity are closely associated with bond order. We performed a correlation analysis to assess the activity relationship between the compounds and TRPA1; however, due to the limited sample size, our findings are only qualitative and do not possess statistical significance. Therefore, the utility of logP and εLUMO as primary descriptors for the drug screening of C14-PA compounds merits further investigation in subsequent research. The type and number of unsaturated bonds in polyacetylene (PA) compounds are crucial determinants of their biological activity. For instance, C17-PA compounds with at least two alkenes and two acetylenes in a conjugated system display high toxicity [2]. In summary, we suggest that the presence of two triple bonds and one double bond in C14-PA compounds is fundamental to their high activity. The observed activity differences are predominantly associated with molecular flexibility, logP, and electronic properties. These findings could improve the predictive accuracy of three-dimensional quantitative structure–activity relationship (3D-QSAR) models for these compounds. Our preliminary findings and investigations into the mechanisms and structure–activity relationships of polyacetylenes lay a theoretical foundation for research into polyacetylenes, particularly for targeting TRPA1 in disease treatment.

## 4. Materials and Methods

### 4.1. Structure Preparation and Binding Site Prediction

The geometric parameters and three-dimensional configurations of three PA compounds were calculated using the Gaussian-09 package [22] with the B3LYP method and 6-31+g(d) basis set. Three C14-PA small molecules are electrically neutral and achieve stable conformations through energy minimization. We utilized the cryo-EM structure of the ligand-free TRPA1, constructed by Jianhua Zhao et al. (PDB code: 6V9W) [17], and subsequently generated the structural coordinates of rat TRPA1 through a fully automated protein structure homology modeling server, SWISS-Model [23]. Through literature research and the FTMAP server [24], we identified several potential binding sites. Subsequently, ligand docking was performed using AutoDock Vina 1.1.2 [25], with the coordinates of the center grid box determined by the predicted binding sites. A stochastic global optimization of the scoring function was employed to dock the ligands into the preliminary identified possible binding sites. Ten models of the ligand were generated at each predicted site. The selection of ligands was based on affinity and molecular conformation. The flow chart in Figure 3a was utilized for the final determination of the binding sites. The TRPA1 molecular structure graphs were drawn using 3D PROTEIN IMAGING [26].

### 4.2. Molecular Dynamics Simulation and Binding Energy Calculation

Molecular dynamics (MD) simulations and the LIE method were employed to calculate the binding energy for all potential binding sites. The most probable binding sites were identified by assessing the stability of the ligand structure in predicting binding sites and the magnitude of the binding energy. MD simulations were conducted using the entire TRPA1 channel. Small-molecule topologies were generated using an online server from the Automated Topology Builder (ATB) [27]. The topology files for proteins were generated using the Gromacs 2018 package with the Gromos96-53A6 force field. The pH of the protein was set to 7.0 by calculating the pKa values on the PDB2PQR [28] website. MD simulations of complexes were conducted using Gromacs 2018.6 [29]. A membrane environment was constructed by embedding POPC lipids using the InflateGro tool [30]. The SPC explicit solvent was used for solvation. Several Na^+^ and Cl^−^ counter-ions were added to the systems to neutralize charge. Energy minimization was performed using the steepest descent algorithm for 1500 steps, followed by a 100 ps isochoric–isothermal (NVT) simulation at 300 K and a 100 ps isothermal–isobaric (NPT) simulation at 1 atm, with all heavy atoms restrained with a force constant of 1000 kJ/(mol·nm^2^). Finally, the positional restraints were released, and the production MD simulation was run for 200 ns with a time step of 2 fs. For simulation parameters, the systems were constrained using the LINCS algorithm [31], and waters were constrained using the SETTLE algorithm. Short-range interactions were calculated with a cutoff of 1.2 nm, while long-range electrostatic interactions were computed using the Particle Mesh Ewald (PME) summation method [32].

In this study, the absolute binding free energy was computed using the widely adopted Linear Interpolation of Energy (LIE) method [33]. According to the LIE method, Δ*G_bind_* is assumed from differences (Δ*V^ele^* and Δ*V^vdw^*) in the ensemble-averaged electrostatic <$V_{lig-sur}^{Ele}$> and van der Waals interaction energies <$V_{lig-sur}^{Vdw}$> involving the ligand and its environment, as obtained from simulations of its protein-bound and unbound states in the free state (water). The parameters α and β in Equation (1) were used as LIE parameters to evaluate Δ*V^ele^* and Δ*V^vdw^*, and γ is an optional offset parameter. The Δ*G_bind_* of the ligand to the protein is given in Equation (1), where α is 0.18 [34]. The chemistry of the ligand determines the value of β, which is set to 0.43 in the electroneutral compound without hydroxyl and 0.37 in the electroneutral compound with hydroxyl [31,32]. A constant term γ was introduced to obtain the absolute binding free energy. γ is a hydrophobic characteristic parameter, and the same value of γ was adopted for all three complexes, by regression fitting γ = −4.066 Kcal/mol [35]. The Lennard-Jones term and Coulomb potential energy term were extracted from the molecular dynamics trajectory stabilization stage. The binding free energy was calculated using the gmx energy and gmx lie modules within the Gromacs2018 package. Over recent years, the LIE method has been successfully applied to calculate the binding free energy of various protein–ligand complexes, yielding good results [36,37,38,39]. The experimental binding energy (${\Delta G}^{EXP}$) was calculated using Equation (2), where (R) and (T) represent the gas constant and experimental temperature, respectively.

(1) $$\Delta G_{bind}=\alpha \Delta V^{vdw}+\beta \Delta V^{ele}+\gamma$$

(2) $${\Delta G}^{EXP}\approx RT{InIC}_{50}$$

### 4.3. Interaction Analysis

Weak interactions between ligands and proteins, such as electrostatic forces, hydrogen bonding, steric repulsion, and van der Waals (vdW) forces, are commonly observed [40]. Given the limitations of static complex structures in providing comprehensive information, we resorted to dynamic analysis, employing typical configurations obtained from simulations. A subsequent cluster analysis was conducted on the ligand and selected configurations. The ligand was then fixed in place for a 1 ns dynamics simulation, yielding 1001 frames of trajectories. The Multiwfn 3.8 [41] software was utilized to analyze the average reduced density gradient (aRDG) [42]. The aRDG was obtained by averaging the density gradient ($\bar{\Delta \rho (r)}$) and density ($\bar{\rho (r)}$) of 1001 frames according to Equation (3). The aRDG results were visualized using VMD (Visual Molecular Dynamics) [43].

(3) aRDG(r)=12(3π2)13Δρ(r)¯ρ(r)43¯

### 4.4. Molecular Property Calculation and Correlation Analysis

To explore the relationship between the physical and chemical properties of the three PA compounds and their activity values, the following properties were calculated for each compound: the lowest unoccupied molecular orbital (LUMO), highest occupied molecular orbital (HOMO), ionization potential (VIP), electron affinity (VEA), log P, lipole, and virtual log P. The virtual log P, Broto log P, and lipole of all molecules were evaluated using VegaZZ 3.2.1 software [44]. The Map of Lipophilicity Potential (MLP) and electrostatic potential surfaces (EPSs) were generated using VegaZZ and GaussView 5.0.9 software, respectively. VIP and VEA were calculated using Equations (4) and (5), respectively.

VIP = E(N − 1) − E(N)(4)

VEA = E(N) − E(N + 1)(5)

The lowest unoccupied molecular orbital (LUMO), highest occupied molecular orbital (HOMO), ionization potential (VIP), and electron affinity (VEA) of the three PA compounds were calculated using the QM/MM program gmx2qmmm [45] and the stable conformations from MD. In this calculation, the three PA compounds were designated as the QM region, with a computational level of B3LYP/6-311*+g(2d,p). The MM region consisted of the protein molecule, with MM atoms represented by the point charge field (PCF) as input to the QM calculation. Our objective was to determine the electronic properties of the three PA compounds in the background charge environment of the protein. The correlation between molecular properties and activity values was derived using KNIME built-in nodes. Specifically, the correlation matrix was generated through the linear correlation node. The linear correlation model was obtained from the Weka (Linear Regression3.7) node, with attribute selection methods based on the M5 method [46].

## 5. Conclusions

In this section, we identified site-II, the C14-PA binding site, which is situated near the A-loop region. This region is characterized by a cluster of nonpolar residues that form a hydrophobic binding pocket. Notably, only Echinophorin B is capable of stabilizing the upward conformation of the A-loop. Binding free energy calculations indicate that the interactions between PAs Echinophorin D, Echinophorin B, and Echinophorin A with TRPA1 are predominantly influenced by van der Waals (vdW) interactions. A decomposition analysis of small-molecule fragments reveals that the binding affinity of two triple bonds and one double bond is strikingly strong, consistent with dynamic interaction calculations. In the binding conformation, Echinophorin D and Echinophorin B display similar positioning and orientation. In contrast, Echinophorin A, characterized by the branched-chain ends of carbon–carbon single bonds, exhibits a distinct conformational direction due to its enhanced flexibility. Furthermore, our study evaluates the lipophilicity and electronic properties of the three polyacetylene (PA) compounds. Echinophorin A exhibits a high logP value, while Echinophorin B possesses a lower logP and relatively stable electronic properties. Correlation analysis reveals that the activity of these compounds against TRPA1 is significantly associated with their logP and εLUMO values, highlighting the critical role of these properties in their interaction dynamics.

## Figures and Tables

**Table 1:** LIE average calculation of absolute free energy of complex (KJ/mol) at site-Ⅱ. D is Echinophorin D. B is Echinophorin B. A is Echinophorin A. P for proteins, L for ligands.

Ligand	[U^el^]_P-L_	[U^vdw^]_P-L_	[U^el^]_L-Rest_	[U^vdw^]_L-Rest_	[U^el^]_L-water_	[U^vdw^]_L-water_	Δ*G*_bind_	ΔG^EXP^	γ/Δ*G*_bind_
Ⅱ-D	−54.2478 ± 2.3	−103.834 ± 2.5	−16.0441 ± 2.8	−19.4748 ± 1.3	−73.246 ± 0.051	−74.9464 ± 0.095	−24.506	−23.320	69.465%
Ⅱ-B	−37.1776 ± 2.9	−82.3549 ± 5.8	−36.6222 ± 1.9	−32.7639 ± 3.7	−72.6516 ± 0.052	−75.9172 ± 0.095	−24.612	−25.439	69.166%
Ⅱ-A	−29.4323 ± 3.0	−133.438 ± 2.2	−33.4841 ± 3.2	−5.97493 ± 1.3	−71.5446 ± 0.045	−77.2402 ± 0.180	−24.566	−24.926	69.295%

**Table 2:** Amino acid residue numbers of three PA compounds interacting with TRPA1. (a) Echinophorin D; (b) Echinophorin B; (c) Echinophorin A.

Residue ID	606	611	613	621	622	623	624	638	663	664	666	667
(a)		GLN	PHE									PRO
(b)			PHE	ARG	CYS	PRO	ILE	LEU	TYR	LEU	CYS	PRO
(c)	TRP	GLN	PHE				ILE					PRO
**Residue ID**	**668**	**669**	**670**	**671**	**677**	**680**	**681**	**682**	**686**	**687**	**690**	**990**
(a)	LEU		MET	THR	THR			TYR				GLU
(b)	LEU					VAL	ILE		THR	ILE	VAL	
(c)		SER				VAL	ILE	TYR				

**Table 3:** The electronic properties of PA compounds in the background charge of the protein.

Molecule	Echinophorin D	Echinophorin B	Echinophorin A
HOMO (eV)	−8.315	−6.543	−7.861
LUMO (eV)	−3.784	−4.166	−4.264
Difference (eV)	4.531	2.377	3.597
VIP (kcal mol^−1^)	223.44	190.688	222.778
VEA (kcal mol^−1^)	55.672	54.917	77.785
Potency EC50 μM	30.9 ± 2.8	25.0 ± 3.0	20.3 ± 3.2

## Data Availability

Data is contained within the article/Supplementary Material.

## Supplementary Materials

The following supporting information can be downloaded at https://www.mdpi.com/article/10.3390/ijms252011290/s1.
