# Supplementary material for: The Interaction Mechanism Between C14-Polyacetylene Compounds and the Rat TRPA1 Receptor: An In Silico Study
Source: Int J Mol Sci. 2024 Oct 20;25(20):11290. doi: 10.3390/ijms252011290 (PMC11508972; doi:10.3390/ijms252011290)
Supplement: Supplementary file 1 [file ijms-25-11290-s001.zip › ijms-3244582-supplementary.pdf]

Supplementary Information for:

Interaction Mechanism Between C14-Polyacetylene Compounds and the Rat TRPA1 Receptor: An In-Silico Study

This document includes:

Figure. S1 Human and rat TRPA1 amino acid sequence. AR12-16 (Ankyrin repeat);S1-S6 (Transmembrane segment);P1-P2 (pore helix 1 and 2);TRPL (TRP-like domain);IFH (Interfacial helix).

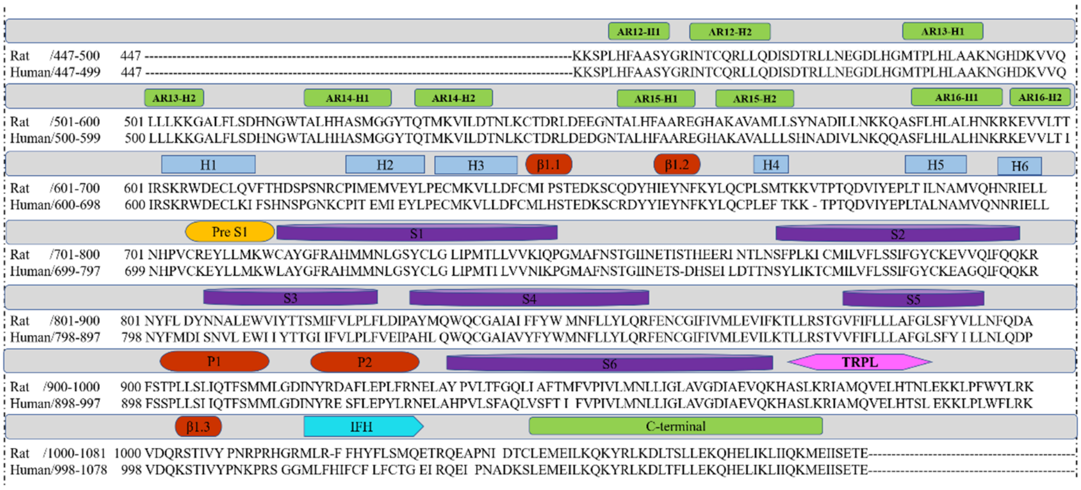

Figure. S2 The RMSD and *D*Gbind of Echinophorin D, Echinophorin B, Echinophorin A and TRPA1 at the binding site changes with time at site-I.

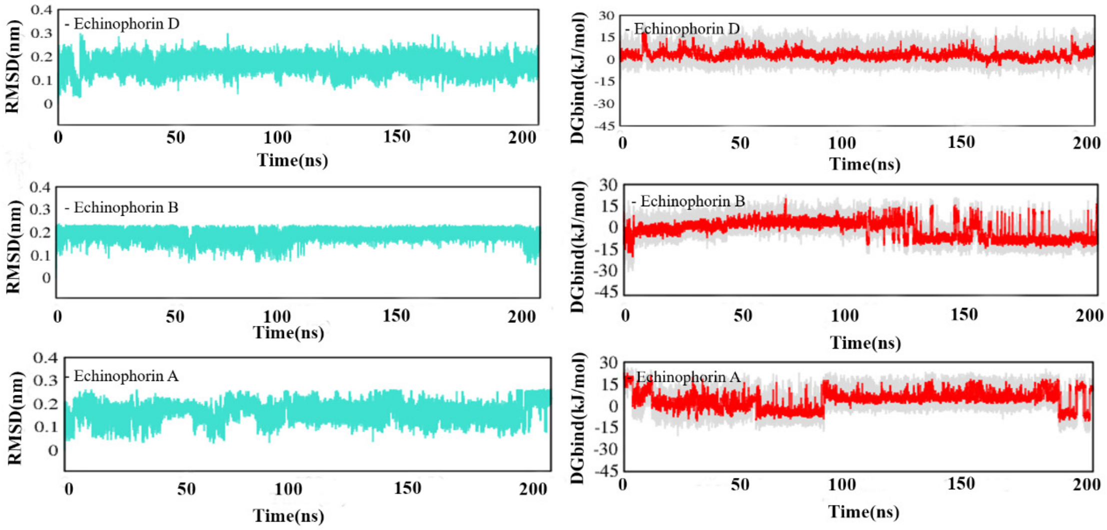

Figure. S3 HOMO, LUMO and EPS of EchinophorinD, Echinophorin B and Echinophorin A.

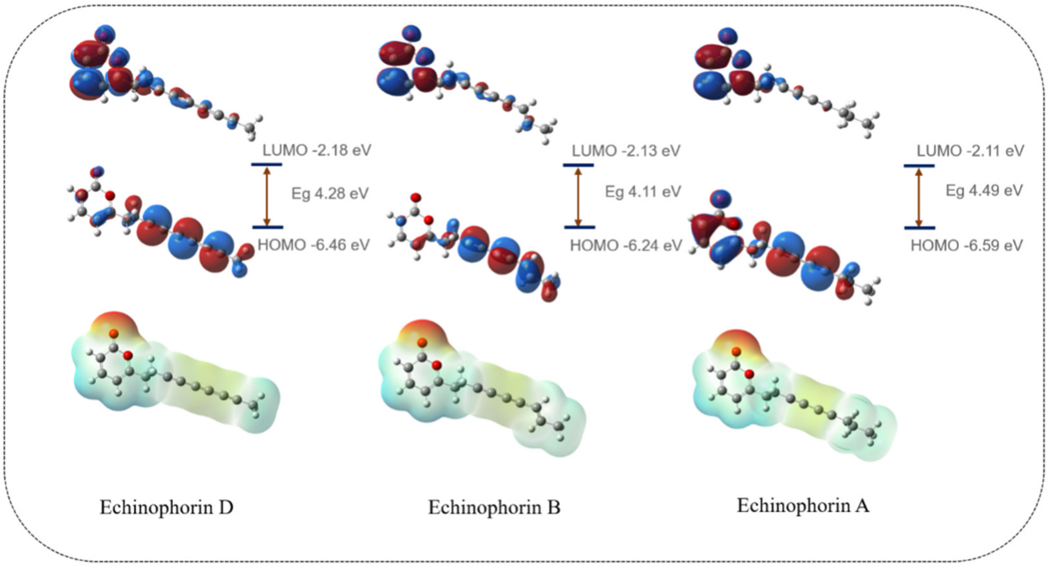

Table S1. Activity of compounds 3–5 on calcium influx in HEK293 cells transfected with rTRPA1.

Compounds	Potency Ec_{50} μM	IC_{50} inh TRPA1μM(AITC 100μM)
Echinophorin D (3)	30.9±2.8	87.0±1.5
Echinophorin B (4)	25.0±3.0	37.2±0.8
Echinophorin A (5)	20.3±3.2	45.7±3.5
